# A Bright and Colorful Future for G-Protein Coupled Receptor Sensors

**DOI:** 10.3389/fncel.2020.00067

**Published:** 2020-03-20

**Authors:** Luca Ravotto, Loïc Duffet, Xuehan Zhou, Bruno Weber, Tommaso Patriarchi

**Affiliations:** ^1^Institute of Pharmacology and Toxicology, University of Zurich, Zurich, Switzerland; ^2^Neuroscience Center Zurich, Zurich, Switzerland

**Keywords:** neurotransmitters, neuromodulators, GPCRs, fluorescent proteins, genetically encoded sensors, *in vivo* imaging

## Abstract

Neurochemicals have a large impact on brain states and animal behavior but are notoriously hard to detect accurately in the living brain. Recently developed genetically encoded sensors obtained from engineering a circularly permuted green fluorescent protein into G-protein coupled receptors (GPCR) provided a vital boost to neuroscience, by innovating the way we monitor neural communication. These new probes are becoming widely successful due to their flexible combination with state of the art optogenetic tools and *in vivo* imaging techniques, mainly fiber photometry and 2-photon microscopy, to dissect dynamic changes in brain chemicals with unprecedented spatial and temporal resolution. Here, we highlight current approaches and challenges as well as novel insights in the process of GPCR sensor development, and discuss possible future directions of the field.

## Introduction

A variety of neurochemicals, including transmitters, modulators, peptides and hormones, are constantly released within the brain during neuronal and glial communication, and play a fundamental role in coordinating physiological brain functions. Tools for monitoring the dynamic changes of individual neurochemicals are therefore highly desirable. For decades fast-scan cyclic voltammetry and microdialysis have been the gold-standard techniques used for measuring the extracellular concentrations of neurochemicals during animal behavior (Kehr and Yoshitake, 2013). However, intrinsic challenges of these analytical chemistry techniques, such the limited molecular specificity and number of detectable neurochemicals for voltammetry (Carter and Shieh, 2010), and poor temporal resolution for microdialysis (Chefer et al., 2009), as well as the low spatial resolution in both systems generated a great need for new technologies capable of bridging these gaps. Specific advantages and limitations of these techniques for monitoring individual neurotransmitters in brain tissue are reviewed in Zeng et al. (2019).

Optical microscopy techniques in combination with genetically encoded sensors are emerging as a powerful solution that can elegantly answer this need. In particular the recently introduced genetically encoded sensors based on a single circularly permuted green fluorescent protein (cpGFP) engineered into G-protein coupled receptors (GPCR), which for simplicity here we refer to as “GPCR sensors,” can enable the detection of neuromodulatory molecules at high resolution in awake behaving animals (Patriarchi et al., 2018; Sun et al., 2018). One of the greatest benefits of these new genetically encoded sensors is their unique ability to detect spatially resolved neuromodulatory signals at high-resolution, which was demonstrated using two-photon imaging in the fly brain (Sun et al., 2018; Handler et al., 2019) and in the mouse cortex (Patriarchi et al., 2018). Exciting new questions in the field can now be addressed thanks to the level of spatial and temporal resolution allowed by these tools, such as whether neuromodulatory signals can be transmitted in a cell-type specific manner or heterogeneously from neuronal projections.

**FIGURE 1:**
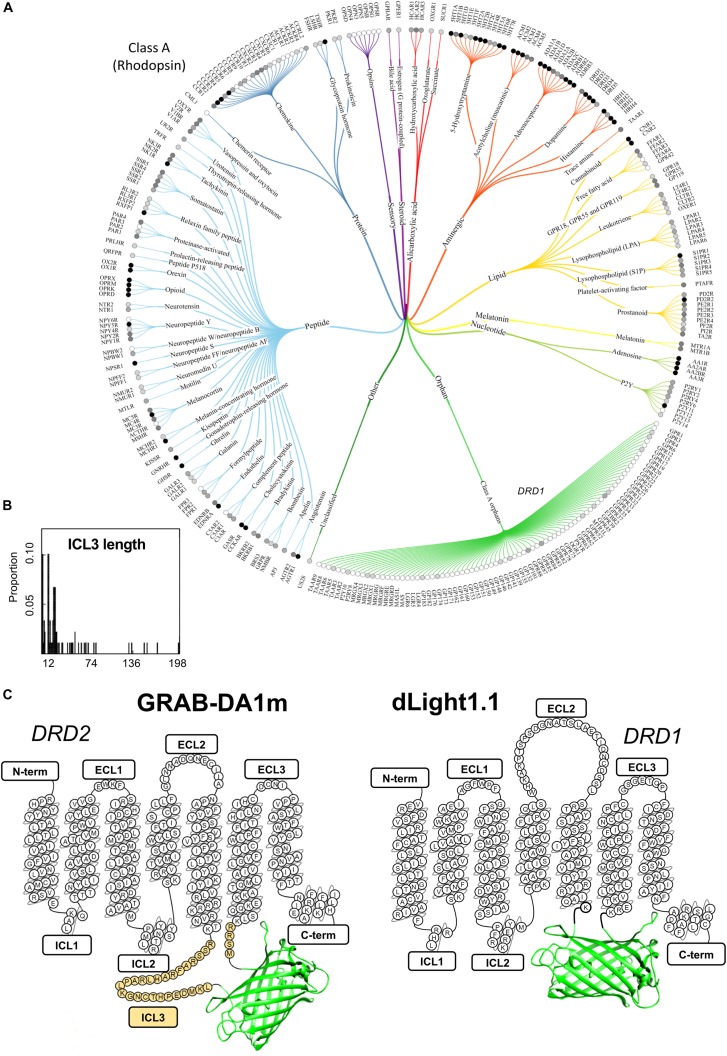
A snapshot of the current GPCR sensor engineering landscape. **(A)** Class-A GPCR family tree. Receptors are grouped and branches are color-coded based on ligand type. Gray-scale circles in front of the receptor name represent the number of currently available ligands (white: 0, light gray: >100, gray: >500 and black: >1000). Reproduced from Pándy-Szekeres et al. (2018). **(B)** Length distribution of ICL3 (region where cpGFP is inserted during the sensor engineering process) for several class-A GPCRs highlights the high degree of variability in this region among different receptors. The number of ICL3 aminoacid residues is shown on the x axis, while the relative probability of each length from a total of 89 analyzed GPCRs is shown on the y axis. Reproduced with permission from Otaki and Firestein (2001). **(C)** Schematic depiction of representative members of the two classes of genetically encoded dopamine sensor classes developed to date: dLight1.1 and GRAB-DA1m. Aminoacid residues of sensor sequence belonging to the original dopamine receptors (DRD1 and DRD2) are shown in the snake-plot. The insertion position of circularly permuted green fluorescent protein (cpGFP) is indicated for both sensor types. N-term, N-terminus; ICL, intracellular loop; ECL, extracellular loop; C-term, C-terminus. Third intracellular loop, ICL3, is highlighted in yellow.

These tools purposely combine the high ligand-binding affinity and molecular specificity which were fine-tuned in the receptor by natural evolution, with the large sensitivity typical of intensity-based probes engineered from cpGFP (Marvin et al., 2011; Chen et al., 2013b; Marvin et al., 2013; Kostyuk et al., 2019). Because GPCRs are a very large family of receptors (class-A alone comprises approximately 350 members without including odorant receptors (Pándy-Szekeres et al., 2018), for an overview see Figures 1A,B), in principle they represent a largely unexplored pool from which novel fluorescent sensors could be developed to probe a vast amount of endogenous neurochemicals. Although FRET or BRET-based biosensors of GPCR activation have been available for a long time (Vilardaga et al., 2003; Sleno et al., 2016), to date only a few GPCR sensors have been introduced for *in vivo* sensing of dopamine (Patriarchi et al., 2018; Sun et al., 2018), acetylcholine (Jing et al., 2018, 2019a), and norepinephrine (Feng et al., 2019). The ability to reveal the intimate spatial and temporal details of neurotransmitter release in living animals is only possible with this new type of sensors and not with previously available FRET or BRET-based probes, due to their limited dynamic range (Jing et al., 2019b). Thus, the innovative field of GPCR sensor development represents a true technological breakthrough and, while still in its early days, is likely to continue expanding to cover many more neurochemical ligands as well as toward bright and colorful new directions.

## Current Strategies and Bottlenecks in GPCR-Sensor Development

The first examples of GPCR sensors were only recently introduced when two similar but independent engineering approaches were published almost simultaneously (Patriarchi et al., 2018; Sun et al., 2018). These studies produced the two alternative genetically encoded dopamine sensor families named dLight1 and GRAB-DA1. The general concept behind both approaches is similar: inserting cpGFP at specific locations between the transmembrane helix 5 (TM5) and 6 (TM6) of a human dopamine receptor is used as a mean to generate sensitive fluorescent reporters of receptor conformational change. Due to their genetically encoded nature these sensors can be easily expressed in living animals, and because conformational motion at the interface of TM5 and TM6 is a classical feature of GPCR activation and reflects ligand binding (Venkatakrishnan et al., 2013), their fluorescent signals can be used as a proxy of endogenous dopamine dynamics.

When compared side by side the approaches taken to develop the two classes of sensors have important differences. In dLight1 cpGFP completely replaces the third intracellular loop (ICL3) as well as small portions of TM5 and TM6 of the D1 dopamine receptor (DRD1) (Patriarchi et al., 2018), while in GRAB-DA1 it is inserted within the ICL3 of the D2 dopamine receptor (DRD2), and a large stretch of 30 aminoacids is carried over from the original ICL3 (Figure 1C; Sun et al., 2018). Similar to GRAB-DA1 sensors, the approaches taken for developing both the acetylcholine and norepinephrine sensors also relied on the presence of a variable amount of ICL3 residues surrounding cpGFP (Jing et al., 2018; Feng et al., 2019).

In all cases, the final goal is to maximize the coupling between receptor conformational change and the fluorescence response of cpGFP. This process, builds on previous knowledge established for genetically encoded sensors of similar design (Chen et al., 2013b; Marvin et al., 2013) and mostly involves the generation of libraries where the linker regions connecting GPCR to cpGFP are subject to sequence randomization and the resulting sensor variants are individually screened for their fluorescence response.

Strikingly, the sensor design used in the development of dLight1, which is completely devoid of ICL3 residues, was demonstrated to be quite versatile. In fact, the simple grafting of a “fluorescent protein module” (i.e., cpGFP flanked by short linkers derived from the dopamine sensor) into other GPCR subtypes made it possible to rapidly generate preliminary sensors for norepinephrine, serotonin, melatonin, and opioid neuropeptides (Patriarchi et al., 2018). Although these prototype probes may not sensitive enough for *in vivo* application, they may represent an ideal starting point for further optimization through targeted mutagenesis at a reduced number of sites, and thus could provide a useful shortcut in a process that is otherwise very costly and time-consuming. Considering that the ICL3 is an extremely variable region among different class-A GPCRs (Figure 1B) both in terms of size and composition (Otaki and Firestein, 2001; Unal and Karnik, 2012), and that this region is predicted to be intrinsically disordered, based on sequence composition (Jaakola et al., 2005), the presence of a considerable portion of the ICL3 in GRAB-DA1 is likely to prevent the versatile grafting of the fluorescent protein module for rapid engineering of novel GPCR sensors.

The concept of versatile engineering is somewhat reflected in the field of GPCR crystallography, where long and disordered ICL3 sequences are counterproductive and are thus commonly replaced with small fusion proteins (e.g., T4 lysozyme; Thorsen et al., 2014), or stabilized with the aid of nanobodies (Manglik et al., 2017). We believe that, in order to be most effective, future engineering efforts should follow a “semi-rational” approach where the starting point for cpGFP insertion into the TM5-TM6 interface of a GPCR is set by the receptor sequence (e.g., the positively charged residues identified during the development of dLight1; Patriarchi et al., 2018) and site-directed mutagenesis screening follows. In this regard, a deeper understanding of the relationship between the receptor, linker, and fluorescent protein components of the sensor, which could be obtained by structural studies. For instance a sensor structure obtained by cryogenic electron microscopy would provide important information on the specific orientation of charged, polar and hydrophobic residue sidechains at the GPCR/cpGFP interface, which could guide future sensor optimization efforts. The current lack of structural information for both dLight1 and GRAB-DA1 sensors, makes it very difficult to achieve a clear understanding of how the receptor conformational change is capable of triggering the fluorescence of cpGFP, and to elucidate the role that linker regions and the residual ICL3 residues play in this process.

Overall, every screening approach in GPCR sensor development faces the same obstacle: a large number of variants to be screened and the low throughput of current mammalian cell-based screening assays. To overcome this hurdle possible strategies could make use of a recently established screening assay, combining Fluorescence-Activated Cell Sorting (FACS) with robotic cell-picking, which was successfully used to evolve voltage sensors derived from integral membrane proteins (Piatkevich et al., 2018). However, implementation of a robotic arm for cell-picking may not be easily applicable in many labs due to the inherent technical complexity of the system. As an alternative, we envision that in the future it may become advantageous to devise novel FACS-based screening methods, perhaps in combination with microfluidic channels that can cyclically integrate two independent cell-sorting steps. Such innovative assay concepts could in theory enable selection of sensor variants based on fluorescence in both an active and inactive state from a library pool of 10^6^–10^9^ cells, which would ideally suit GPCR sensor development and dramatically increase the scale of the process.

Although all attention of current GPCR sensor screening approaches focuses on obtaining sensors with the largest maximal fluorescence changes, this property is not by itself sufficient to guarantee success when testing the probe in the living brain. The apparent affinity of the probe is another important factor to be considered when imaging neuromodulators. Owing to its design each probe can only reliably work within a narrow concentration range. Because the levels of release for endogenous neuromodulators are variable among different brain regions, mostly depending on the abundance of neuromodulatory projections in the area, multiple probes with complementary affinity ranges need to be developed in order to most sensitively detect the same neuromodulator in different regions. Up to now, sensors with different affinity ranges have been obtained mostly by using different receptor subtypes as a starting point (in the case of dopamine sensors, DRD1, DRD2, DRD4) (Patriarchi et al., 2018; Sun et al., 2018), and in few cases have been tuned by mutagenesis of one specific site in the GPCR moiety (Patriarchi et al., 2018; Feng et al., 2019). Future sensor development could tremendously benefit from computational modeling-based approaches aimed at fine tuning the sensor apparent affinity or even ligand-selectivity (Feng et al., 2017).

## The Opportunity of a Lifetime

A deeper understanding of the basic photophysical properties of GPCR sensors may lead to novel applications based on different fluorescence properties (e.g., fluorescence lifetime) and open new research directions. In fact, while intensity-based fluorescence readouts can provide valuable information about the dynamics of biological systems using simple instrumentation, they suffer from limitations (e.g., wavelength-dependent absorption and scattering, different detector sensitivities or expression dependence for single-wavelength sensors) that make them unsuitable for quantitative measurements of analyte concentrations (Waters, 2009; Yellen and Mongeon, 2015). These drawbacks are particularly critical for deep brain imaging or when comparing different samples or measurements done with different instrumentation. In the case of GPCR sensors, the kinetics of a biological response to stimulation can be reliably accessed (Patriarchi et al., 2018; Sun et al., 2018), but important parameters such as the absolute extent of the response or the baseline levels of the analyte are much more difficult to retrieve. On the contrary, fluorescence lifetime is an intensive rather than extensive physical quantity, which reflects the kinetics of excited to ground state relaxation and is independent on the absolute intensity of the signal (Lakowicz, 2006). This latter factor is critical as it eliminates the effects of laser fluctuations, tissue absorption and detector sensitivity. Also, in a microscopy or fiber photometry setup, the lifetime is much less affected by tissue scattering than intensity (Dowling et al., 1997; Vishwanath et al., 2002).

All these advantages have sparked a growing interest in techniques like fluorescence lifetime imaging (FLIM) (Berezin and Achilefu, 2010; Borst and Visser, 2010; Chen et al., 2013a) or fluorescence lifetime fiber photometry (FLiP) (Brown et al., 1994; Saxl et al., 2011; Lagarto et al., 2019; Lee et al., 2019a, b), which have been successfully applied to obtain more quantitative information on analyte concentrations (Díaz-García et al., 2017; Melo et al., 2017) or protein interactions (Yasuda et al., 2006; Sun et al., 2011; Walther et al., 2011). Recent studies investigated the optimization and application of fluorescent protein (FP) pairs to produce FLIM sensors based on fluorescence resonance energy transfer (FLIM-FRET) (Visser et al., 2010; George Abraham et al., 2015; Martin et al., 2018). The process of FRET introduces an additional path for the excited state decay of a “donor” fluorophore, due to the transfer of excitation energy to an “acceptor.” Due to the relationship between quantum yield and lifetime of a fluorophore (Lakowicz, 2006; Noomnarm and Clegg, 2009), the effect of FRET on lifetime is particularly straightforward (the higher the FRET efficiency, the shorter the donor lifetime) and the design of FLIM-FRET sensors is, at least in first approximation, conceptually simple.

**FIGURE 2:**
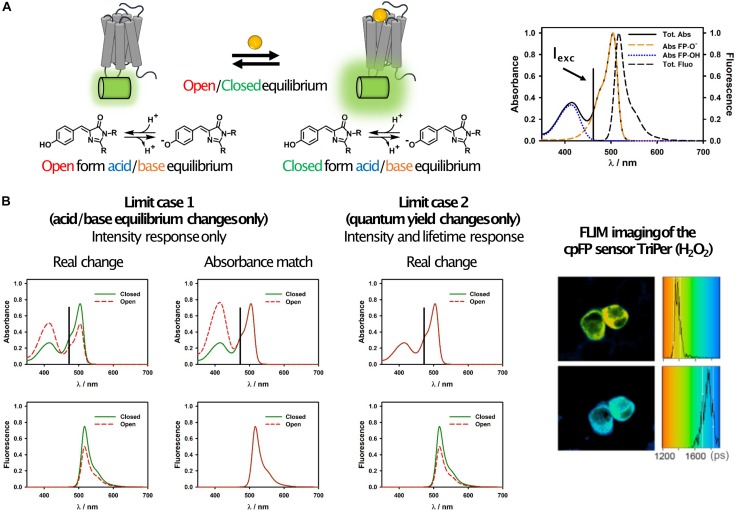
Simplified photophysical description of cpFP sensors with emphasis on FLIM. **(A)** Schematic representation of the equilibrium occurring in GPCR sensors (left) and of their spectroscopic properties (right). It can be observed how the absorption spectrum is actually the sum of two bands, one due to the protonated (acid) form of the chromophore and one to the deprotonated (basic) one. The latter is also the only emissive form in most cpFPs used in sensors. This is certainly true if the sensor is excited at longer wavelengths, as shown in these examples, where only the basic form absorbs. **(B)** Representative spectral changes for the two limit cases described in the text (left), and FLIM images and histograms of RINm5f cells expressing the cpFP sensor TriPer before (right, top) and after (right, bottom) exposure to 0.2 mM H_2_O_2_ (reproduced without modifications from Melo et al., 2017; under Creative Commons license: http://creativecommons.org/licenses/by/4.0/). Possible candidates for FLIM can be identified by looking at the changes of the absorption and fluorescence spectra of the sensors upon analyte binding. If only the acid/base equilibrium changes, but not the quantum yield of the basic form, then no lifetime change is expected (limit case 1). An easy way to conceptualize this phenomenon is that if the absorbance of the open and closed forms at the excitation wavelength would be the same (i.e., if the two would absorb the same number of photons), then their fluorescence intensity would also be the same (column “absorbance match”). On the other hand, if there are changes in the fluorescence intensity but not in the acid/base equilibrium (limit case 2), then changes in the quantum yield are occurring and will likely (but not necessarily) result in a lifetime change. We note that this is a simplified scheme which takes into account only a subset of the photophysical processes occurring in cpFP sensors, thus: (a) unexpected results may occur by strictly following this simplified scheme as a general rule for all sensors, (b) lifetime changes can occur also as result of other mechanisms.

On the other hand, the field of FLIM sensors based on circularly permuted fluorescent proteins (cpFPs) is still largely underdeveloped. While some groups have reported the use of cpFP sensors with FLIM readout (Tantama et al., 2011; Mongeon et al., 2016; Melo et al., 2017; Díaz-García et al., 2019), the sensor characterization has been mostly limited to empirical calibrations, and no clear guidelines for the choice of FPs that would give optimal lifetime changes currently exist. In our opinion, this is largely due to the complexity of the problem. While in FRET sensors a single photophysical process is responsible for the observed changes, in cpFPs a complex combination of effects needs to be taken into account (Barnett et al., 2017; Molina et al., 2019). In a cpFP sensor, the acid and basic forms of the chromophore exist in equilibrium. Each form has its own absorption spectrum and fluorescence quantum yield (usually, only the basic form has a high quantum yield and is defined as “emissive” form), and the equilibrium concentrations can change upon opening and closing of the protein barrel (Figure 2A). Interconversion between the two forms can happen also in the excited state (Meech, 2009; Barnett et al., 2017), so that the excitation of the acid form can result in the emission of the basic one. In the case of multiphoton absorption, also the absorption cross-sections can depend on the barrel conformation (Molina et al., 2019). As a consequence of this complexity, the relation between intensity and lifetime is very difficult to predict.

Ignoring for simplicity excited-state dynamics and multiphoton effects, the photophysical basis of sensor function can be described by two limit cases (Figure 2B). In the first one, the changes in the fluorescence intensity of the sensor are due exclusively to changes in the relative abundance of the acid-base equilibrium forms of the chromophore upon analyte binding, while the quantum yield of the basic form (the emissive one) remains unaffected (Figure 2A). In this scenario, while fluorescence intensity changes can be large (and wavelength dependent), no change in lifetime is expected. In the second limit case, there are no changes in the acid-base equilibrium upon analyte binding, but the fluorescence quantum yield of the basic form changes. While this is not always the case, very often variations in fluorescence quantum yield are accompanied by variations in lifetime (Lakowicz, 2006). Real world scenarios lie in between these two extremes, and thus FLIM sensors with smaller or larger (and more or less wavelength-dependent) lifetime changes can in principle be developed based on these considerations.

Careful photophysical studies (Barnett et al., 2017; Molina et al., 2019) have shown that even for seemingly similar cpFP sensors the mechanisms that determine the fluorescence response can change considerably, and that good candidates for FLIM definitely exist. We expect that in the future more studies will be devoted to this specific aspect and cpFPs optimized for FLIM readouts will start to emerge. This will in turn boost the field of GPCR-based sensors, paving the way toward a more quantitative understanding of the role of neurochemicals in brain functions. Particularly for physiological dynamics that do not manifest themselves in fast and quasi-binary transients (as is often the case for calcium signals), quantification of molar concentrations of the target molecule is essential. This will allow for reliable comparisons among different brain areas, different animals or data acquired by different labs, and for analyzing data obtained in chronic long-term imaging over days and months.

## The Push Toward Red-Shifted Wavelengths

It is reasonable to imagine that in the near future the development of GPCR sensors will also expand toward both red and near-infrared (NIR) wavelengths, by taking advantage of a large variety of fluorescent proteins available at these wavelengths (Rodriguez et al., 2017). This has for instance been the case for genetically encoded calcium sensors, which have recently been developed at near-infrared wavelengths based on mIFP (Qian et al., 2019) or GAF-FP (Subach et al., 2019). Such GPCR sensors would benefit from inherent advantages of using red-shifted light for excitation, such as increased penetration depth and lower phototoxicity. They would also increase the quality and quantity of potential applications. For instance, the availability of red-shifted GPCR sensors would enable “mix and match” approaches where green and red sensors for two different neurotransmitters could be combined in dual-color fluorescence imaging, similar to what has been previously achieved for red calcium sensors (Dana et al., 2016). This approach could be used for instance to resolve long-standing challenges, such as the unequivocal proof of co-release between two neurotransmitters. A prominent example of this is the case of dopamine and norepinephrine: previous studies imply that the two catecholamines can be synthesized and simultaneously released by the locus coeruleus (Kempadoo et al., 2016; Takeuchi et al., 2016), however, direct evidence for this is lacking as well as the important information of whether this phenomenon is constitutive or regulated.

The landscape of raw materials for building GPCR sensors is constantly evolving, as new fluorescent and chromoproteins are added to the plate (Lambert et al., 2019). Development of GPCR sensors based on near-infrared fluorescent proteins could in principle expand the solution space for multiplex imaging, and allow us to image up to three different aspects of neural communication and activity simultaneously. In addition, the NIR window (650–900 nm) offers improved optical transmission properties in living animals because of the relatively lower absorption component of endogenous molecules such as hemoglobin or melanin (Ntziachristos et al., 2003). We predict that the development of GPCR sensors absorbing in this wavelength range, that could for instance be engineered based on bacterial phytochromes (e.g., BphP1; Yao et al., 2016), will enable successful application of the probes with optoacoustic imaging, a scalable imaging modality capable of resolving whole-brain activity three-dimensionally at millisecond time scale and 100 μm resolution non-invasively (i.e., without the need for optical fiber or gradient index lense implantation) (Gottschalk et al., 2019). Another advantage of red/NIR FPs is that their photophysical properties are optimal for three-photon absorption (3PA) in the 1700 nm optical window (Horton et al., 2013; Deng et al., 2019), which is recently emerging as a superior technique for deep *in vivo* imaging (Horton et al., 2013; Miller et al., 2017). Due to the much reduced scattering and absorption, 3PA at these wavelengths is limited only by signal-to-noise ratio (which depends on protein brightness) up to more than 3 mm depth (Horton et al., 2013), and structural imaging of neurons has already been demonstrated at a depth of 1.4 mm (Horton et al., 2013), reaching the subcortical region of the mouse brain. Especially if combined with FLIM detection (Ni et al., 2019), that eliminates artifacts due to absorption and scattering, 3PA at 1700 nm would constitute a promising strategy for non-invasive imaging of neurochemicals in deep brain regions, while at the same time improving the cell resolution ability in densely labeled samples with respect to two-photon absorption (Ouzounov et al., 2017).

## Discussion

Considering the important and multifaceted roles that neurochemicals play in the nervous system, the thirst for novel sensor types capable of probing these molecules in their natural environment is likely to continue increasing. Although GPCR sensors are an elegant technological solution, some caveats need to be considered. One example is the potential interference with endogenous neuromodulatory signaling of target cells, which has not yet been extensively characterized in living animals. Our hope is that, with time, a careful evaluation of the side-effects of GPCR sensor expression will elucidate best experimental practices that can allow us to make the most out of these tools without significantly altering the system under investigation. For a more in-depth discussion of the limitations of these tools please refer to our previously published protocols (Patriarchi et al., 2019). All things considered, we envision a bright future for GPCR sensor development, with important achievements to be expected along at least three directions. First, the continuous improvement of existing GPCR sensors (in terms of increased dynamic range, basal brightness, kinetics, photostability, etc.) will follow an analogous path to that of the GCaMP family of calcium sensors (Dana et al., 2019), and will build upon the continuous discovery and directed evolution of novel and improved variants of fluorescent proteins and chromoproteins. Second, in striking difference with calcium sensors, the optical measurement of the actual concentrations of neurochemicals *in vivo*, rather than merely their dynamic behavior, is a much needed task. The demonstration that GPCR sensors can be utilized in quantitative techniques such as FLIM and the design of specifically optimized sensors is still lacking and will likely be a central topic in the field in the near future. Third, the need for multiplexed imaging will not only spur the design of novel GPCR sensors to probe an increasing number of neurochemicals, but also the diversification of existing sensors in terms of emission wavelengths. The latter will dramatically expand the possibilities to simultaneously image multiple neurochemicals, or observe how their dynamics correlate with other important phenomena such as calcium or metabolite transients. While the path ahead for this new class of sensors certainly presents its challenges, some of which were highlighted in this work, their unique features and wide applicability will keep us and others motivated to develop new and improved variations which will clearly contribute to shape a bright and colorful future for neurochemical imaging.

## Author Contributions

TP and LR wrote the manuscript with contributions from XZ, LD, and BW.

## Conflict of Interest

TP was co-inventor on a patent application (WO/2018/098262A1) for the technology described in this paper. The remaining authors declare that the research was conducted in the absence of any commercial or financial relationships that could be construed as a potential conflict of interest.
